# The Aryl-Hydrocarbon Receptor Protein Interaction Network (AHR-PIN) as Identified by Tandem Affinity Purification (TAP) and Mass Spectrometry

**DOI:** 10.1155/2013/279829

**Published:** 2013-12-05

**Authors:** Dorothy M. Tappenden, Hye Jin Hwang, Longlong Yang, Russell S. Thomas, John J. LaPres

**Affiliations:** ^1^Department of Biochemistry and Molecular Biology, Michigan State University, East Lansing, MI 48824-1319, USA; ^2^Center for Integrative Toxicology, Michigan State University, East Lansing, MI 48824-1319, USA; ^3^Center for Mitochondrial Science and Medicine, Michigan State University, East Lansing, MI 48824-1319, USA; ^4^The Hamner Institutes for Health Sciences, Research Triangle Park, NC 27709, USA

## Abstract

The aryl-hydrocarbon receptor (AHR), a ligand activated PAS superfamily transcription factor, mediates most, if not all, of the toxicity induced upon exposure to various dioxins, dibenzofurans, and planar polyhalogenated biphenyls. While AHR-mediated gene regulation plays a central role in the toxic response to dioxin exposure, a comprehensive understanding of AHR biology remains elusive. AHR-mediated signaling starts in the cytoplasm, where the receptor can be found in a complex with the heat shock protein of 90 kDa (Hsp90) and the immunophilin-like protein, aryl-hydrocarbon receptor-interacting protein (AIP). The role these chaperones and other putative interactors of the AHR play in the toxic response is not known. To more comprehensively define the AHR-protein interaction network (AHR-PIN) and identify other potential pathways involved in the toxic response, a proteomic approach was undertaken. Using tandem affinity purification (TAP) and mass spectrometry we have identified several novel protein interactions with the AHR. These interactions physically link the AHR to proteins involved in the immune and cellular stress responses, gene regulation not mediated directly via the traditional AHR:ARNT heterodimer, and mitochondrial function. This new insight into the AHR signaling network identifies possible secondary signaling pathways involved in xenobiotic-induced toxicity.

## 1. Introduction

Dioxins, a family of toxic chemical compounds that are highly stable and readily bioaccumulate, have been the focus of extensive research for several decades [1, 2]. Investigations have shown that these compounds can arise naturally from forest fires and cooking; however, industrial processes, such as paper bleaching and pesticide manufacturing, produced dioxins in large quantities. This increased production has lead to pervasive environmental contamination. In fact, over 500,000 tons of soil and sediment are contaminated with 2,3,7,8 tetrachlorodibenzo-p-dioxin (TCDD) in the US alone [3].

TCDD and other dioxins induce a host of toxic responses in mammals, such as thymic involution, immunosuppression, wasting syndrome, and chloracne. The aryl-hydrocarbon receptor (AHR) mediates virtually all of these dioxin-induced pathologies [4, 5]. Studies revealed that dioxins serve as ligands which activate the AHR [6], a member of the basic helix-loop-helix-Per-Arnt-Sim (bHLH-PAS) superfamily of transcription factors [7]. In the absence of ligand, the AHR can be found in the cytosol bound to a dimer of the heat shock protein of 90 kDa, Hsp90, and the immunophilin-like protein, AIP (also known as XAP2 and ARA9) [8, 9]. Upon ligand binding the AHR translocates to the nucleus and binds its heterodimeric partner, the aryl-hydrocarbon receptor nuclear translocator (ARNT) protein. The AHR/ARNT dimer regulates expression of a battery of genes involved in metabolism and elimination, including xenobiotic metabolizing enzymes *CYP1A1 *and* CYP1B1 *[10, 11]. The translocation and subsequent DNA binding of the AHR is necessary for TCDD-induced toxicity; however, the toxicity associated with dioxin exposure does not directly correlate with the levels of the AHR found in the various tissues [12–16]. In fact, the liver, an organ with relatively high AHR expression, is fairly resistant to TCDD-induced toxicity [17]. This tissue-specific response raises the possibility that accessory factors and secondary signaling are involved in modulating the receptor's ability to promote toxicity.

Besides Hsp90 and AIP, the AHR also has been shown to interact with p23, Cdk4, the retinoblastoma protein (Rb), and RelA of the NF*κ*B complex [16, 18–20]. Early reports demonstrated that p23 was a core AHR complex protein. The p23 protein has been reported to function in numerous cellular roles including organelle function, DNA repair, and cell mobility [21]. Recently, p23 was shown to specifically interact with the N-terminus of Hsp90 [22]. Other reports have shown its interaction with the AHR to be transient and nonessential for the receptor's function [23, 24]. The interactions with Cdk4, Rb, and RelA link the receptor to cell cycle, kinase signaling, and gene regulation not exclusively mediated by the AHR. Moreover, the AHR has been implicated in estrogen and glucocorticoid receptor signaling and developmental processes [25–27]. Taken together, this multifaceted interaction network suggests that AHR biology is complex and capable of impacting several cellular responses.

The extent of the AHR protein interaction network (AHR-PIN) has not been addressed on a proteomic level in a mammalian system. To investigate the role that protein-protein interactions have in AHR-mediated dioxin toxicity, we have established the AHR-PIN using tandem affinity purification (TAP) and mass spectrometry (MS). Here we report the AHR-PIN as assembled from MS data collected in the presence and absence of TCDD ligand, using a Hepa1c1c7 cell line with stable overexpression of an AHR-TAP-tagged construct [28]. The Hepa1c1c7 cell line is extensively used in AHR research and well characterized in its responses to TCDD exposure. Although the liver is not considered highly sensitive to TCDD-induced toxicity, the tissue does experience marked changes in gene expression, hyperlipidemia, and hepatosteatosis [29]. Defining the AHR-PIN in this cell line offers insights to how TCDD influences AHR biology and induces toxicity in this tissue type. Identified AHR interactors include mitochondrial, cell cycle, immune response, and transcription factor proteins. The new AHR-PIN has identified different secondary pathways that could potentially influence TCDD-induced AHR-mediated toxicity. Previously, we reported AHR's influence on mitochondrial function through its interaction with the ATP5*α*1 subunit of the ATP synthase complex [28]. Here we focus on the receptor's interaction with another mitochondrial protein, mitochondrial ribosomal protein L40 (Mrpl40).

## 2. Materials and Methods

### 2.1. Materials

Oligonucleotides were synthesized at the Macromolecule Synthesis Facility at Michigan State University. The pTarget and pGEM-T Easy vectors were purchased from Promega (Madison, WI) and pZome1C vector was obtained from Cellzome (Cambridge, UK). Antibodies used in these experiments were obtained from the following resources; rabbit polyclonal anti-AHR antibodies were a generous gift from Dr. Christopher Bradfield (University of Wisconsin-Madison), rabbit anti-Mrpl40 (cat no. HPA006181) was purchased from Sigma (St Louis, MO), mouse anti-COX4 (cat no. A21348) was purchased from Life Technologies (Grand Island, NY), goat anti-rabbit (cat no. sc2004), goat anti-mouse (cat no. sc2005), normal rabbit IgG (cat no. sc2027) and protein G resin were obtained from Santa Cruz (Santa Cruz, CA). All other chemicals used in these experiments were reagent grade and purchased from Sigma Aldrich (St. Louis, MO).

### 2.2. Plasmid Constructs and Cell Culture

The cDNAs for the murine AHR and the green fluorescent protein (GFP) were amplified using the following primers.

AHR: 5′-ggatccccaccatgagcagcggcgccaacatcacc-3′ and 5′-ggatcctgcactctgcaccttgcttagg-3′, GFP: 5′-gggggatccaccatggtgagcaagggcgac-3′ and 5′-gtggatccccgggcccgcggtaccgtcgactgc-3′. The amplicons were subcloned into pZome1C vector placing the TAP-tag at the C terminal of both the AHR and GFP genes. Ampicillin resistance was used for clonal selection and each positive clone was sequence verified. Phoenix-eco and Hepa1c1c7 cells were cultured in DMEM high glucose media with L-glutamate and supplemented with 10% cosmic calf serum (CCS), 100 units/mL penicillin, 100 *μ*g/mL streptomycin, and 1 mM sodium pyruvate. HepaC12 cells were cultured in DMEM high glucose media w/ L-glutamate and supplemented with 10% cosmic calf serum (CCS), and 1 mM sodium pyruvate. Tissue culture media and supplements were obtained from Life Technologies and CCS was obtained from HyClone (Waltman, MA). Hepa1c17 TAP-AHR and TAP-GFP were grown in the media described above and puromycin (2 *μ*g/mL, US Biological, Swampscott, MA) was used for selection.

### 2.3. Transfection/Stable Cell Line Infection

The retroviral vectors, AHR-TAP and GFP-TAP, were transfected into Phoenix-eco cells with Lipofectamine 2000 (Life Technologies) via manufacturer's instructions. After incubation (5 to 8 hours, 37°C) in the presence of DNA, the media were changed to Phoenix cell growth media containing chloroquine (25 *μ*M). Cells were then incubated for 24 hours (37°C). After incubation, cells were given fresh Phoenix cell growth media and incubated for an additional 24 hours (32°C). The media were collected after incubation; centrifugation was used to remove cellular debris (3 mins, 45 ×g, in RT7 Sorvall, Rockford, IL), and the media were purified using a 0.45 *μ*m membrane filter (Millipore). Virus containing media was placed on Hepa1c1c7 wild type target cell lines and incubated for 3 hrs at 32°C and 5% CO_2_. Media containing 15 *μ*g/mL polybrene were then added to target cells and incubated for 24 hours (32°C). After incubation, fresh media were added to target cells and incubated (37°C) until plates reached 80% confluence. Cells were passaged and selected using puromycin (2 *μ*g/mL).

### 2.4. Western Blot Analysis

Tissue culture samples were harvested and total protein concentration was determined as previously described [30]. Proteins samples were separated on Nu-Page Bis-Tris 4–12% gradient gels, transferred to nitrocellulose membranes, and probed with assorted antibodies as previously described [31]. Western blots were visualized using Pierce (Rockford, IL) ECL western blotting substrate.

### 2.5. Tandem Affinity Purification

**Figure 1:**
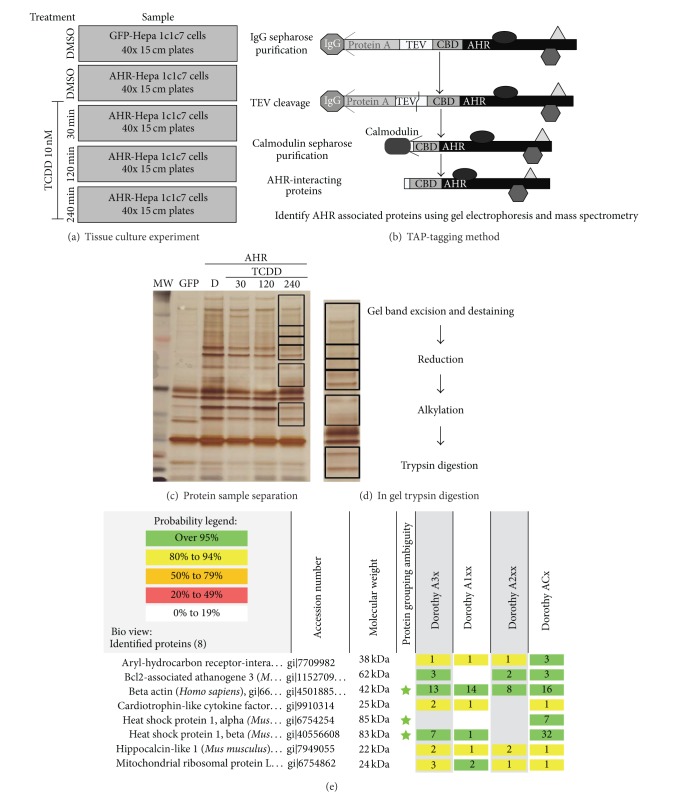
Experimentation overview. (a) Schematic outline of TCDD exposure experiments in Hepa1c1c7 cell lines. GFP and AHR samples were treated with vehicle (DMSO (D), 0.01%); the remaining three AHR samples were treated with TCDD (10 nM) for the labeled time (30, 120, and 240 mins). (b) Schematic of the tandem affinity purification (TAP) protocol. After TCDD exposure, cell lysate samples underwent two rounds of purification utilizing the TAP methodology. AHR and GFP TAP-tagged proteins and their associated complexes were isolated and then separated using gel electrophoresis. (c) Representative image of gel replicates for final TAP eluate samples separated in 4–12% Nu-page gel matrix. Boxes around the areas of the AHR-TAP 240 min TCDD dose sample lane denote the areas of the gel that were excised from each of the sample lanes. (d) Schematic of the in-gel trypsin digestion protocol. Excised gel samples were destained, and proteins underwent reduction, alkylation, and trypsin digestion. (e) Sample of MS analysis data set. Lastly, the protein fragment samples then underwent MS analysis as described in Section 2.

An estimated 2.5 × 10^9^ Hepa1c1c7 TAP-AH and TAP-GFP cell were challenged with DMSO (vehicle control, 0.01%) or TCDD (10 nM) for 30, 120, or 240 minutes in 3 independent experiments (Figure 1(a)). Media were removed and cells were washed three times in cold phosphate buffered saline (PBS). Cells were harvested in TAP-tag lysis buffer (TTLB, 5% glycerol, 50 mM Tris, pH 7.5, 50 mM MgCl_2,_ 100 mM NaCl, 0.1% NP40, 1 mM DTT, 1 mM Na_3_VO_4_, 25 mM NaF, and protease inhibitor tablets) and lysed by two cycles of freeze/thaw, and insoluble material was removed by centrifugation (7000 ×g, 20 mins). Next, samples were incubated with 200 *μ*L of IgG Sepharose beads (GE Healthcare, Waukesha, WI) for 4 hrs, at 4°C and rotation. Beads were collected by centrifugation (45 ×g, 30 sec) and transferred to Poly-Prep Chromatography columns (BioRad, Hercules, CA). Beads were extensively washed with TAP-tag lysis buffer and then with TEV cleavage buffer (10 mM Tris, pH 7.5, 150 mM NaCl, 0.1% NP40, 0.5 mM EDTA, and 1 mM DTT). IgG beads were incubated (18 hrs, 4°C) in 1.5 mL TEV cleavage buffer containing AcTEV protease (450 units). Eluates were collected by gravity flow. Beads were washed with 1.5 mL of TEV buffer and 9 mL of calmodulin binding buffer (CBB) (10 mM *β*-mercaptoethanol, 10 mM Tris, pH 7.5, 150 mM NaCl, 1 mM MgOAc, 1 mM imidazole, 0.1% NP40, and 2 mM DTT). Eluate and washes were combined. Samples were then incubated with 200 *μ*L calmodulin affinity resin (Stratagene, La Jolla, CA) (4 hrs, 4°C). After incubation, calmodulin resin was transferred to new Ploy-Prep Chromatography columns, and resin was washed extensively with CBB. Warm 3x SDS buffer (4.8% SDS, 100 mM Tris pH 6.8, 16% glycerol, 8% *β*-mercaptoethanol, and 0.4% bromophenol blue) was used to elute protein complexes from resin (Figure 1(b)).

### 2.6. Gel Electrophoresis and Mass Spectrometry

Samples were separated on a 4–12% Bis-Tris gradient gel (NuPage, Invitrogen) by electrophoresis. SilverSNAP staining kit (Pierce, Rockford, IL) was used to visualize proteins according to manufacturer's protocol and the gel was photographed (Figure 1(c)). Specific protein bands were excised from the gel matrix, destained with SilverSNAP destaining kit (Pierce, Rockford, IL), and subjected to in-gel tryptic digestion as previously described [32] (Figure 1(d)). The extracted peptides were then automatically injected by a Waters nanoACQUITY Sample Manager (Milford, MA) loaded for 5 minutes onto a Waters Symmetry C18 peptide trap (5 *μ*m, 180 *μ*m × 20 mm) at 4 *μ*L/min in 5% acetonitrile/0.1% formic acid. The bound peptides were eluted onto a Waters BEH C18 nanoACQUITY column (1.7 *μ*m, 100 *μ*m × 100 mm) over 35 minutes with a gradient of 2% B to 35% B in 21 min, 90% B from 21–24 min, and back to constant 5% B at 24.1 min using a Waters nanoACQUITY UPLC (buffer A = 99.9% water/0.1% formic acid, buffer B = 99.9% acetonitrile/0.1% formic acid) with an initial flow rate of 600 nL/min, ramping to 700 nL/min at 80 min and back to 600 nL/min at 86 min. Eluted peptides were sprayed into a Thermo Fisher LTQ Linear Ion trap mass spectrometer outfitted with a MICHROM Bioresources ADVANCE nanospray source. The top five ions in each survey scan are then subjected to data-dependent zoom scans followed by low energy collision induced dissociation (CID) and the resulting MS/MS spectra are converted to peak lists using BioWorks Browser v 3.3.1 (Thermo Fisher, Rockford, IL) using the default LTQ instrument parameters. Peak lists were searched against all mouse sequences available in the NCBI nr database, downloaded on November 16, 2008, from NCBI, using the Mascot searching algorithm, v2.2 (http://www.matrixscience.com/). The Mascot output was then analyzed using Scaffold (http://www.proteomesoftware.com/) to probabilistically validate protein identifications using the ProteinProphet computer algorithm (Figure 1(e)).

### 2.7. Coimmunoprecipitation

Wild type Hepa1c1c7 cells were grown to 80% confluence. Media were removed and cells were washed with ice cold PBS and then harvested with TTLB. Cellular supernatants (500 *μ*g) were incubated with normal rabbit IgG (2 *μ*g/*μ*L) or an anti-AHR antibody (2 *μ*g/*μ*L) for 90 minutes. Following incubation, protein G beads (30 *μ*L) were added to the samples and incubated for an additional 90 minutes. Beads were collected by centrifugation. The supernatant was removed, and the beads were washed extensively in TTLB. Samples were eluted from the beads with warm 3X SDS buffer and separated on a Nu-PAGE Bis-Tris 4–12% gradient gel matrix, transferred to nitrocellulose membrane, and probed with primary AHR or Mrpl40 antibodies.

### 2.8. Cellular Fractionation

Wild type Hepa1c1c7 and AHR null HepaC12 cell lines were treated with vehicle (0.01% DMSO) or TCDD (10 nM) for 6 hrs. After treatment cells were harvested using fractionation buffer (25 mM sucrose, 20 mM Tris-HCl, 1 mM EDTA, pH7.4). The cells underwent one round of freeze/thaw and then dounce homogenized (100 stokes). Insoluble materials were removed by centrifugation (400 ×g, 10 min) and the supernatant was transferred to a new tube. An aliquot of this supernatant was taken as the whole cell lysate (WLC) fraction. Separation of the supernatant and organelle fractions was performed by centrifugation (4,500 ×g, 10 min). The supernatant was removed and an aliquot was taken as the cytosolic fraction (Cyto). The organelle pellet was resuspended in 1 mL of fractionation buffer and dounce homogenized (14–20 strokes). An aliquot of the pellet after resuspension and homogenization was taken as the impure mitochondrial fraction (MF1). Large debris was removed from the MF1 sample by centrifugation (400 ×g, 10 min). The supernatant was transferred to a new tube and mitochondria were isolated by centrifugation (4,500 ×g, 10 min). The mitochondrial pellet was resuspended in 100 *μ*L fractionation buffer and is the pure mitochondrial fraction (MF2).

## 3. Results

### 3.1. Workflow for Proteomic Screen

Each replicate of the proteomic screen was processed following the workflow described in Figure 1 and detailed in the materials and methods section.

### 3.2. Identification of AHR Interactors

TAP was performed on DMSO treated GFP-TAP and AHR-TAP cells and AHR-TAP Hepa1c1c7 samples treated with TCDD (10 nM) for different times (30, 120, 240 minutes), followed by MS analysis of the TAP final eluates. The cytosolic partners of the AHR, Hsp90a, Hsp90b, and AIP were identified in the AHR-TAP sample at a greater than 95% confidence. The identification of the core AHR cytosolic complex proteins demonstrates that this method is a valid means to investigate novel AHR protein interactions.

**Figure 2:**
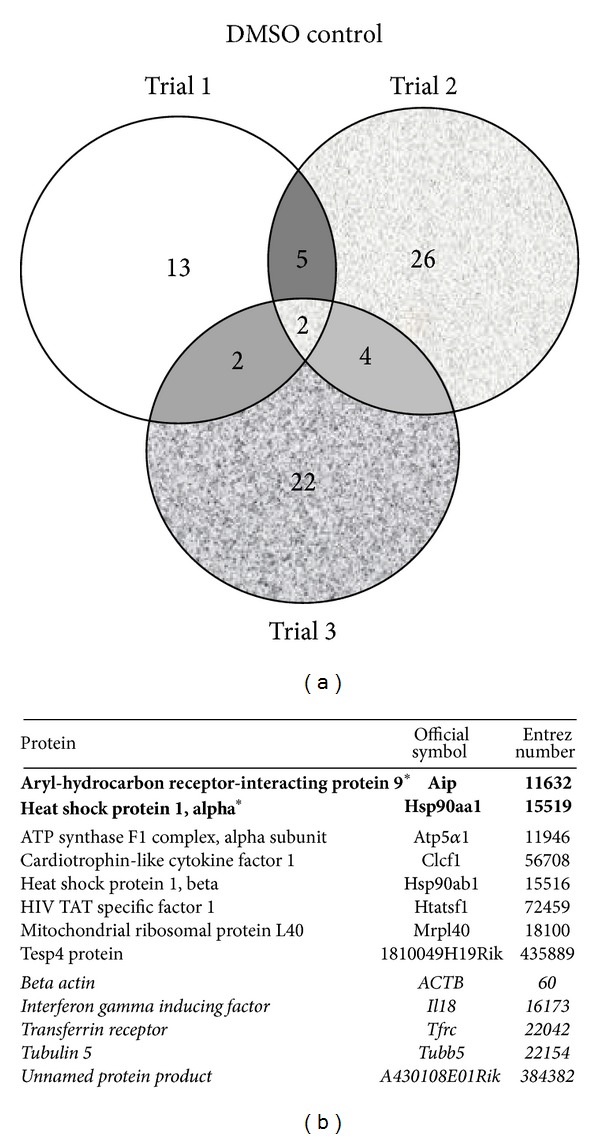
DMSO control data set. (a) Venn diagram of proteins identified by mass spectrometry in 3 data sets of AHR-TAP DMSO (0.01%) control. (b) Table listing of proteins identified in two out of three data sets. Bold type with * denotes a protein represented in all three data sets. Italicized type denotes nonspecific proteins identified in both AHR-TAP and GFP-TAP samples.

**Figure 3:**
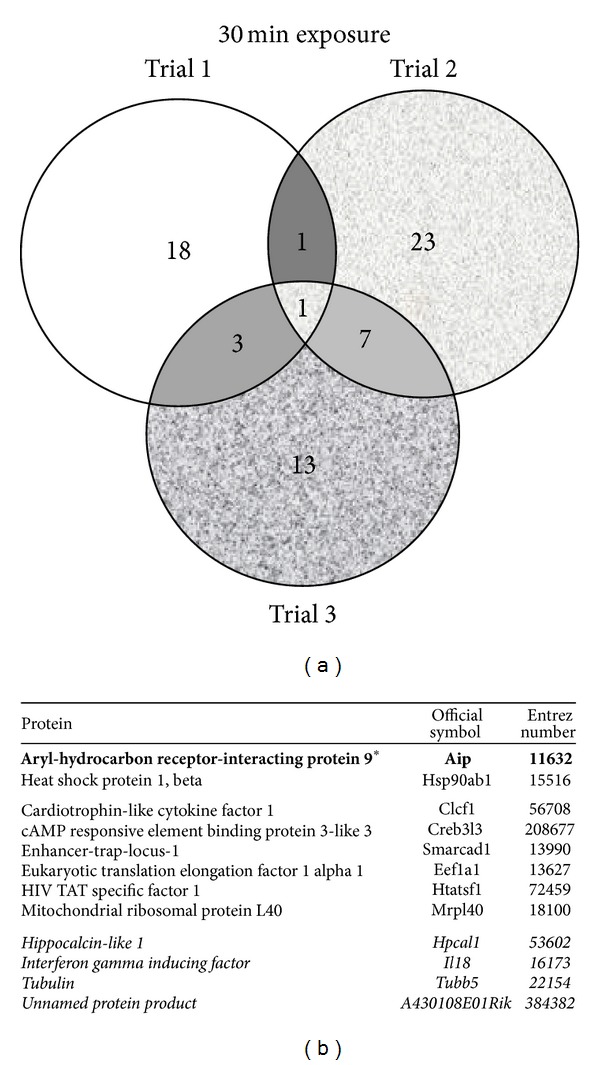
TCDD 30-minute treatment data set. (a) Venn diagram of proteins identified by mass spectrometry in 3 data sets of AHR-TAP 30 min TCDD (10 nM) dosing. (b) Table listing of proteins identified in two out of three data sets. Bold type with * denotes a protein represented in all three data sets. Italicized type denotes nonspecific proteins identified in both AHR-TAP and GFP-TAP samples.

**Figure 4:**
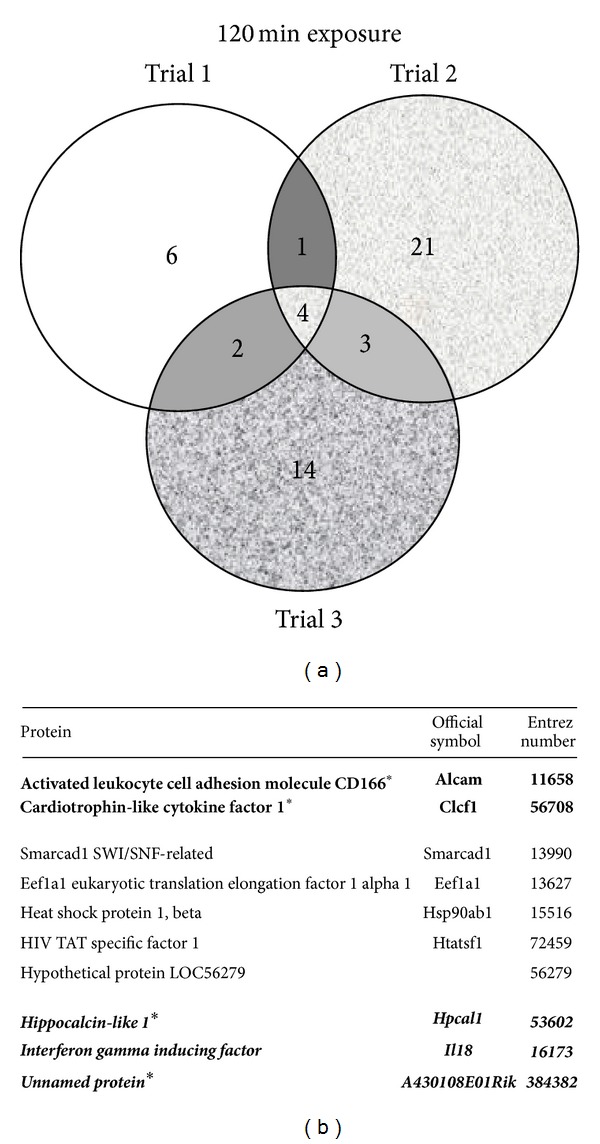
TCDD 120-minute treatment data set. (a) Venn diagram of proteins identified by mass spectrometry in 3 data sets of AHR-TAP 120 min TCDD (10 nM) dosing. (b) Table listing of proteins identified in two out of three data sets. Bold type with * denotes a protein represented in all three data sets. Italicized type denotes nonspecific proteins identified in both AHR-TAP and GFP-TAP samples.

**Figure 5:**
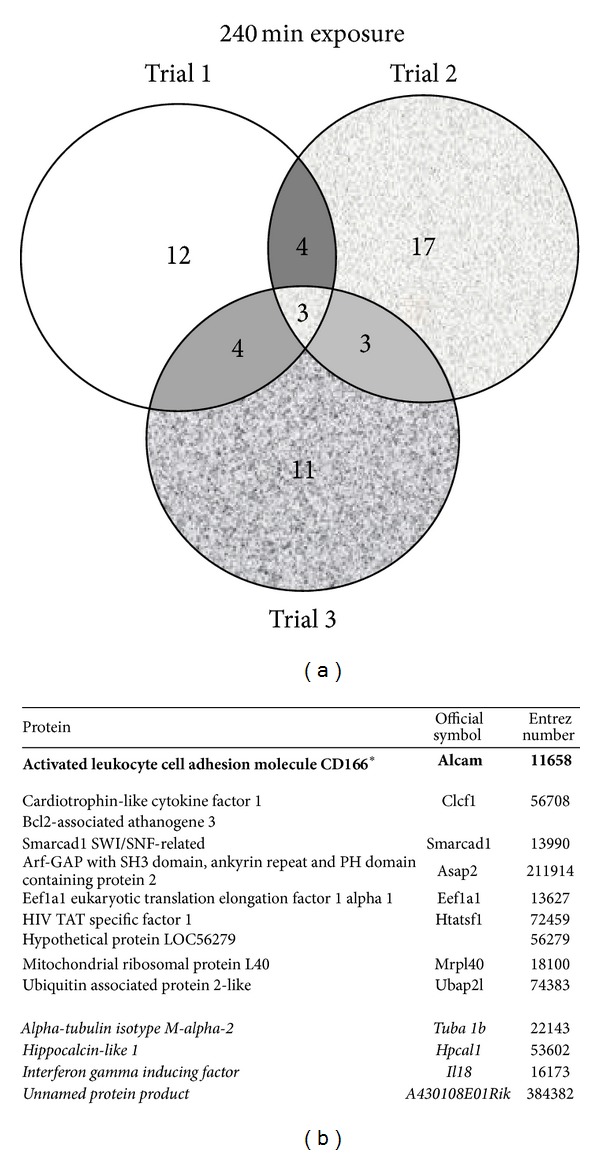
TCDD 240-minute treatment data set. (a) Venn diagram of proteins identified by mass spectrometry in 3 data sets of AHR-TAP 240 min TCDD (10 nM) dosing. (b) Table listing of proteins identified in two out of three data sets. Bold type with * denotes a protein represented in all three data sets. Italicized type denotes nonspecific proteins identified in both AHR-TAP and GFP-TAP samples.

TCDD and vehicle treated AHR-TAP Hepa1c1c7 samples from three separate experiments, per treatment and time point, were analyzed for AHR protein interactors. The GFP-TAP vehicle treated sample data sets were used as a negative control. The TAP samples were separated into discreet bands in a gel matrix and excised for MS analysis (Figure 1(c)). The breakdown of proteins identified per treatment group is as follows. There are 74 proteins identified in the vehicle treatment group of which 13 were identified in 2 or more replicate samples: 4 novel proteins, 4 known interactors, and 5 nonspecific interactors (Figure 2). The 30-minute TCDD treatment groups contained 66 proteins, including 12 that were identified in 2 or more of the replicates: 6 novel proteins, 2 known interactors, and 4 nonspecific interactors (Figure 3). The smallest protein set, a total of 51 proteins, was identified in the 120-minute TCDD treatment groups. This group contained 10 proteins that were identified in 2 or more replicates: 6 novel interactors, 1 known interactor, and 3 nonspecific interactors (Figure 4). Lastly, the 240 minute TCDD treatment data set contains 59 proteins, of which 14 were identified in multiple replicates: 10 novel interactors, no known interactors, and 4 nonspecific interactors (Figure 5). Here, novel proteins are considered previously unreported AHR interactions; some of these novel proteins were found in more than one data set and will be discussed below. The group of previously reported proteins included Hsp90 isoforms, Hsp90*α* and Hsp90*β*, AIP, and the ATP synthase F1 complex, alpha subunit (ATP5*α*1). Nonspecific interactors were found in both the AHR-TAP and GFP-TAP sample data sets and will not be discussed further. The unique proteins of these data sets are detailed below.

In the DMSO treated AHR-TAP samples two proteins are exclusive to this data set (Figure 2). One is the previously reported ATP5*α*1 subunit of the ATP synthase complex [28]. The other is Tesp4, a ubiquitously expressed homolog of pancreatic trypsin [33]. One protein, Mrpl40, was also identified in the 30- and 240-minute TCDD treatment samples. In yeast, this protein has been shown to play a role in growth rate, mitochondrial protein folding, and mitochondrial function [34, 35]. In humans, the Mrpl40 gene is part of a chromosomal deletion of 22q11 in velo-cardio-facial syndrome (VCFS) and DiGeorge syndrome [36, 37]. The remaining two proteins, cardiotrophin-like cytokine factor 1 (Clcf1) and HIV TAT specific factor 1 (Htatsf1), were identified in this and all three (30, 120, 240 minute) TCDD treatment data sets. Clcf1 is a cytokine, highly expressed in tissues of the immune system, and is an activator of kinase pathways [38, 39]. Htatsf1 is a transcriptional cofactor that regulates expression of the HIV-1 LTR [40].

The 30-minute TCDD treated AHR-TAP samples contained one protein exclusive to this treatment set, the cAMP responsive element binding *protein* 3-like 3 (Creb3l3) (Figure 3). Creb3l3, which is also known as CrebH, functions as an endoplasmic reticulum bound transcription factor responsible for regulating hepatic gluconeogenesis [41]. Two of these proteins, Enhancer-trap-locus-1 (Smarcad SWI/SNF-related) and Eukaryotic translation elongation factor 1 alpha 1 (Eef1a1), were identified in all three of the TCDD treated sample sets. Smarcad functions as a DNA helicase associated with SWI/SNF complexes [42]. Eef1a1 facilitates protein synthesis through tRNA transfer to ribosomal machinery [43]. The remaining three proteins in this sample set, Mrpl40, Clcf1, and HIV TAT, were introduced above.

There were no unique proteins to the 120 min TCDD treated AHR-TAP sample data sets. There are two proteins in common between this and the 240 min TCDD treated data sets, an uncharacterized transcript, hypothetical protein LOC56279, and the activated leukocyte cell adhesion molecule CD166 (Alcam) (Figure 4). Alcam has become widely recognized as a cellular marker for several forms of cancer, including melanoma, squamous cell carcinoma, prostate, breast, colorectal, bladder, esophageal, and ovarian cancers [44–46]. The four remaining proteins of these data sets, Smarcad, Eef1a1, Clcf1, and HIV TAT, have been discussed above.

There were three unique proteins to the 240 minutes TCDD treatment AHR-TAP samples, Bcl2-associated athanogene 3 (Bag3), ArfGAP with SH3 domain, ankyrin repeat and PH domain 2 (Asap2), and ubiquitin associated protein 2-like (Ubap2l) (Figure 5). Bag3, the second mitochondrial associated protein identified, is a cochaperone protein involved in the stress response and disease [47, 48]. Bag3 is considered a prooncoprotein, by inhibiting the apoptotic response through its influence on Bcl-2 family members [49, 50]. Asap2, also known as development and differentiation enhancing factor 1 (Ddef1), is an Arf-GTPase activating protein [51, 52]. Ubap2l is associated with proteasome mediated protein degradation and has been implicated in infertility [53]. The seven remaining proteins identified in these data sets were introduced in the previous data sets.

### 3.3. Coimmunoprecipitation Verification of Identified Interactors

**Figure 6:**
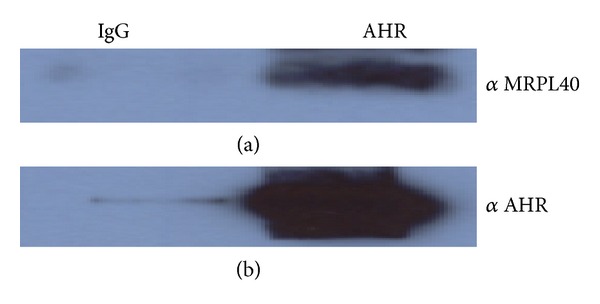
Independent protein interaction verification. Coimmunoprecipitations (Co-IP) using anti-AHR or normal rabbit IgG as negative control were performed in wild type Hepa1c1c7 cells. (a) Western blot probed with anti-MRPL40 (Invitrogen). (b) Positive control, western blot probed with anti-AHR. MRPL40 and AHR control all show marked enrichment in the AHR pulldown lanes over the IgG control lane.

Independent verification of the interactions between the AHR and select proteins identified by mass spectrometry was performed by coimmunoprecipitation in wild type Hepa1c1c7 cells. Similar to previously reported verification of the AHR:ATP5*α*1 interaction [28], this method was used to investigate the interaction between the AHR and Mrpl40 (Figure 6). Mrpl40 was chosen to further investigate the direct link between the AHR and mitochondrial function that was recently reported [28]. Mrpl40 showed marked enrichment in the AHR Co-IP samples over the normal mouse IgG control. These interactions were reproducible in at least two of the three trials performed. The functional consequence of these interactions is currently under investigation.

### 3.4. TCDD Influence on MRPL40 Cellular Localization

**Figure 7:**
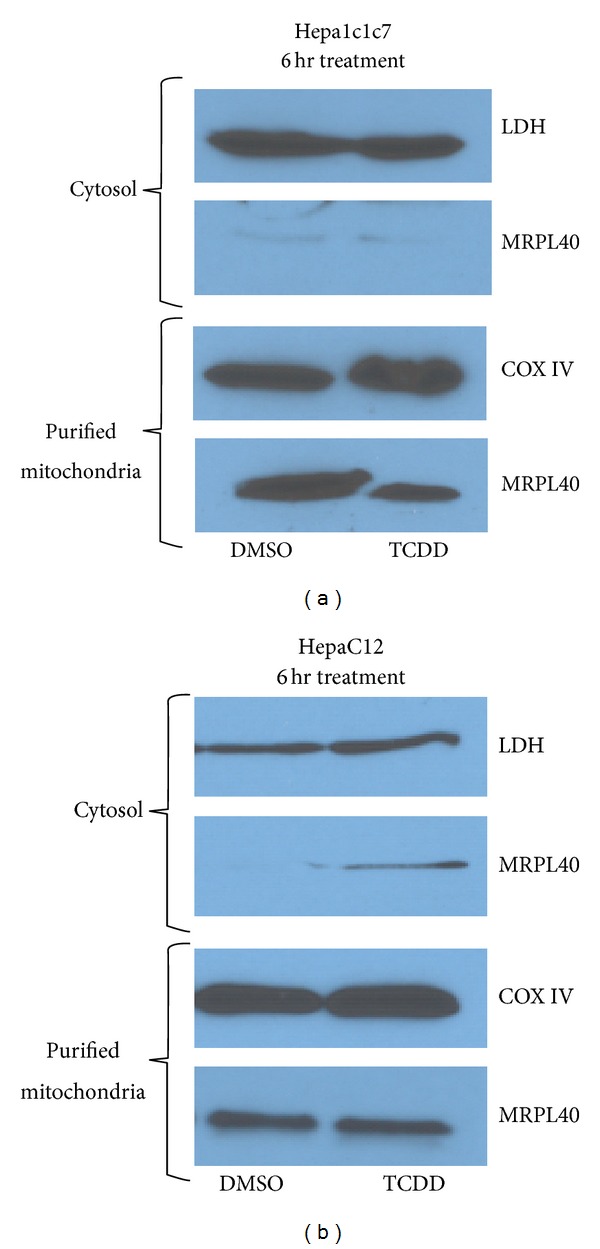
TCDD treatment influences Mrpl40 mitochondrial localization. (a) Cellular fractionation was performed on AHR in wild type Hepa1c1c7 cells. Fractions from cells exposed to TCDD for 6 hours were probed with antibodies specific to MRPL40, lactate dehydrogenase (LDH), and cytochrome c oxidase subunit 4 (COXIV). The results demonstrate a decreased level of mitochondrial localized Mrpl40 following TCDD exposure. (b) Similar cellular fractionations were next performed on AHR null Hepac12 cells. No decrease in Mrpl40 was observed after exposure to TCDD for 6 hours. LDH was used as the cytosolic fraction control protein and COXIV was used as the purified mitochondrial fraction control protein.

We previously reported that TCDD exposure decreased the pool of AHR in the mitochondrial but did not alter ATP5*α*1 levels [28]. Given AHR's interaction with MRPL40, we wanted to determine if TCDD-induced depletion of mitochondrial localized AHR impacted the cellular localization of MRPL40. To investigate this, cellular fractionation was performed on wild type Hepa1c1c7 cells treated with TCDD (10 nM) and vehicle (DMSO) for 6 hrs (Figure 7(a)). A marked decrease in mitochondrial MRPL40 levels was observed in the 6 hr TCDD treatment samples. Next we examined the localization of Mrpl40 in the AHR null cell line, HepaC12. The AHR null cell line does not experience a decrease in mitochondrial Mrpl40 levels in the presence of TCDD (Figure 7(b)).

## 4. Discussion

The AHR's functional relevance is centered on sensing environmental pollutants and regulating developmental processes. Identifying interacting partners for the AHR, therefore, is important to our understanding of the receptor-mediated changes in signaling pathways involved in these processes. The research detailed here represents the first comprehensive analysis of the AHR-PIN in a mammalian system using proteomic technology. The AHR-PIN reveals physical interactions with proteins involved in several cellular processes and disease states with which the receptor has been previously linked. These include cell cycle, apoptosis, immune response, mitochondrial function, and cancer [16, 54–58]. In addition to AHR mediated gene regulation, this data suggests the receptor's potential influence on cellular biology through protein:protein interactions.

A 2004 study conducted using *S. cerevisiae* yeast as a model system reported 54 genes which influence AHR biology [59]. Interestingly, we have identified proteins that demonstrate some correlation with this data. In yeast, SNF12 and SWI3 were shown to influence the AHR and here we report a protein interaction between the AHR and SMAD-CAD, a helicase linked to the SWI/SNF complex. In addition, similar to the identified yeast genes encoding kinases and GTPases, we identified several proteins involved in kinase signaling, including Asap2 and Clcf1. While these similarities are not direct correlations, they do provide another layer of evidence to the complexity of AHR biology and the possible mechanisms involved in AHR mediate toxicity and cellular function.

There are previously reported AHR protein interactions that were not observed here that are of note. The cytosolic complex protein p23 was not identified in any of our MS data as stated earlier. Given the transient nature of its interaction with the AHR its absence is not wholly unexpected. We also did not identify Rb or RelA in any of the data sets. These interactions are also of a transient nature, making identification difficult and we cannot rule out cell type-specific interactions [19, 60]. Moreover, it is possible that the level of these proteins' interaction with the AHR is below the detection limit of this methodology as well. Interestingly, we did identify Cdk4 in some of our MS data; however, this result was not consistent across multiple data sets, but the findings correlate with earlier reports of AHR:CDK4 crosstalk [16]. Finally, the AHR's nuclear partner, ARNT, was detected in various TCDD samples across the dosing time course. The ARNT detected, however, did not meet our threshold cutoff (>80% confidence in two or more samples) in a given time point to be included in the tables. This is most likely due to the inherent variability in the assay and the transient nature of the interaction.

The absence of these known interactors and the variability in the data sets were also noted. This type of variability is not unexpected given that many of the proteins were near the limit of detection of the methodology. It is difficult to determine significant differences between samples as one operates closer to the limit of detection [61]. This increased variability might also be involved in the transient identification of some of the identified interactors. For example, Mrpl40 was identified in every sample except the 120-minute one. This might be biologically important or it might be due to the inherent variability in the methodology and the limitations, in terms of sample size and ability to detect low abundant proteins. This experimental variability might also be confounded by the overexpression system used in this screen. It is difficult, however, to determine the extent to which overexpression has influenced the observations. In addition, the transient nature and complex sample handling protocol will also induce significant amounts of variability between samples. For data analysis of novel AHR protein interactions, we relaxed the confidence level to 80% cutoff. This was done to include proteins that were identified at a greater than 95% confidence in at least one replicate experiment but not all replicates. It should be noted that the variability seen in this screen is consistent with previous published reports for 14-3-3 and c-Myc TAP experiments [62, 63]. Finally, given the inherent variability in this type of analysis, it is as important to investigate the biological importance of those interactions that occurred in a single sample with high significance as it is to investigate those that occurred in multiple replicates.

Previous studies have linked TCDD exposure to mitochondrial dysfunction, including hyperpolarization of the inner membrane, influence on ATP levels, and ROS production [57, 64, 65]. The AHR:ATP5*α*1 interaction and an AHR-dependent hyperpolarization of the mitochondrial inner membrane that was independent of transcription indicate a role for the AHR in mitochondrial function and cellular energetic [28]. Taken together the novel interaction between the AHR and two other mitochondrial associated proteins are of great interest. The first, Bag3, a prooncoprotein, is involved in the mitochondrial stress response and disease [47, 48]. It has also been shown to inhibit the apoptotic response through interactions with Bcl-2 family members [49, 50]. Moreover, there is evidence that it influences the NF*κ*B pathway through its interaction with p65. The interaction between Bag3 and p65 plays a role in the human immunodeficiency virus type 1 (HIV-1) LTR response to NF*κ*B signaling [66]. The physical interactions between Bag3 and the AHR provide an interesting connection between the AHR and cell cycle regulation, apoptosis, and the NF*κ*B pathway [20]. AHR's interaction with Bag3 observed after the longest TCDD treatment could indicate a mechanism by which activated receptor influences apoptotic signaling cascades. The second, Mrpl40, influences growth rate, mitochondrial protein folding, and mitochondrial function in yeast [34, 35]. It is linked to two diseases in humans, velo-cardio-facial and DiGeorge syndromes [36, 37]. Interestingly, these diseases present with heart defects and immune deficiencies. AHR null mice have been shown to have abnormal heart development. Furthermore, the immune system is one of the more sensitive to TCDD-induced toxicity [67–69]. We have currently focused our attentions on the AHR:Mrpl40 interactions.

These AHR protein interactions could indicate other roles for the receptor in cellular energy homeostasis and gene regulation not directly mediated by DNA binding. The temporal differences in the AHR interactions with the mitochondrial proteins reported here and the ATP5*α*1 after TCDD exposure are of significant interest. The AHR:Mrpl40 interaction appears transiently across TCDD treatments in our MS studies. Cellular fractionation experiments demonstrated a decreased mitochondrial pool of Mrpl40 after TCDD treatment in AHR expressing hepatoma cells. This decrease in mitochondrial Mrpl40 was not observed in the AHR null hepatoma cell line. It is noteworthy that the decreased mitochondrial pool of Mrpl40 is observed at the same TCDD exposure time point, 6 hrs, as we observed the increase in membrane polarization previously reported [28].

The novel interactions reported here demonstrate potential roles for the AHR in signaling pathways which may function in concert with AHR-mediated gene regulation in dioxin induced toxicity. While the physical interactions reported here provide another layer of evidence for the AHR's potential roles in several signaling pathways, disease states, and gene regulation events, the functional relevance of these interactions remains in question. This AHR-PIN presents new avenues of investigation to better define a comprehensive understanding of AHR biology.

## References

[1] Poland A, Glover E (1973). Chlorinated dibenzo p dioxins: potent inducers of *δ* aminolevulinic acid synthetase and aryl hydrocarbon hydroxylase. II. A study of the structure activity relationship. *Molecular Pharmacology*.

[2] Knutson JC, Poland A, Khan MAQ, Stanton RH (1981). 2,3,7,8-tetrachlorodibenzo-p-dioxin: toxicity *in vivo* and *in vitro*. *Toxicity of Halogenated Hydrocarbons*.

[3] Haglund P (2007). Methods for treating soils contaminated with polychlorinated dibenzo-p-dioxins, dibenzofurans, and other polychlorinated aromatic compounds. *Ambio*.

[4] Schmidt JV, Su GHT, Reddy JK, Simon MC, Bradfield CA (1996). Characterization of a murine *Ahr* null allele: involvement of the Ah receptor in hepatic growth and development. *Proceedings of the National Academy of Sciences of the United States of America*.

[5] Poland A, Glover E (1990). Characterization and strain distribution pattern of the murine Ah receptor specified by the *Ah*
^d^ and *Ah*
^b−3^ alleles. *Molecular Pharmacology*.

[6] Pollenz RS, Sattler CA, Poland A (1994). Characterization of the Ah receptor for 2,3,7,8-tetrachlrodibenzo-p-dioxin: use of chemical crosslinking and a monoclonal antibody directed against a 59-kDa protein associated with sterod receptors. *Molecular Pharmacology*.

[7] Robles R, Morita Y, Mann KK (2000). The aryl hydrocarbon receptor, a basic helix-loop-helix transcription factor of the PAS gene family, is required for normal ovarian germ cell dynamics in the mouse. *Endocrinology*.

[8] Carver LA, Jackiw V, Bradfield CA (1994). The 90-kDa heat shock protein is essential for Ah receptor signaling in a yeast expression system. *The Journal of Biological Chemistry*.

[9] Carver LA, LaPres JJ, Jain S, Dunham EE, Bradfield CA (1998). Characterization of the Ah receptor-associated protein, ARA9. *The Journal of Biological Chemistry*.

[10] Nebert DW, Atlas SA, Guenthner TM, Kouri RE, Ts'o POP, Gelboin HV (1978). The Ah locus: genetic regulation of the enzymes which metabolize polycyclic hydrocarbons and the risk for cancer. *Polycyclic Hydrocarbons and Cancer: Chemistry, Molecular Biology and Environment*.

[11] Nebert DW, Roe AL, Dieter MZ, Solis WA, Yang Y, Dalton TP (2000). Role of the aromatic hydrocarbon receptor and [Ah] gene battery in the oxidative stress response, cell cycle control, and apoptosis. *Biochemical Pharmacology*.

[12] Bunger MK, Glover E, Moran SM (2008). Abnormal liver development and resistance to 2,3,7,8-tetrachlorodibenzo-p-dioxin toxicity in mice carrying a mutation in the DNA-Binding domain of the aryl hydrocarbon receptor. *Toxicological Sciences*.

[13] Bunger MK, Moran SM, Glover E (2003). Resistance to 2,3,7,8-tetrachlorodibenzo-p-dioxin toxicity and abnormal liver development in mice carrying a mutation in the nuclear localization sequence of the aryl hydrocarbon receptor. *The Journal of Biological Chemistry*.

[14] Carver LA, Hogenesch JB, Bradfield CA (1994). Tissue specific expression of the rat Ah-receptor and ARNT mRNAs. *Nucleic Acids Research*.

[15] Okey AB (2007). An aryl hydrocarbon receptor odyssey to the shores of toxicology: the deichmann lecture, international congress of toxicology-XI. *Toxicological Sciences*.

[16] Barhoover MA, Hall JM, Greenlee WF, Thomas RS (2010). Aryl hydrocarbon receptor regulates cell cycle progression in human breast cancer cells via a functional interaction with cyclin-dependent kinase 4. *Molecular Pharmacology*.

[17] Swanson HI, Bradfield CA (1993). The AH-receptor: genetics, structure and function. *Pharmacogenetics*.

[18] Hutchison KA, Stancato LF, Owens-Grillo JK (1995). The 23-kDa acidic protein in reticulocyte lysate is the weakly bound component of the hsp foldosome that is required for assembly of the glucocorticoid receptor into a functional heterocomplex with hsp90. *The Journal of Biological Chemistry*.

[19] Ge NL, Elferink CJ (1998). A direct interaction between the aryl hydrocarbon receptor and retinoblastoma protein: linking dioxin signaling to the cell cycle. *The Journal of Biological Chemistry*.

[20] Tian Y, Ke S, Denison MS, Rabson AB, Gallo MA (1999). Ah receptor and NF-*κ*B interactions, a potential mechanism for dioxin toxicity. *The Journal of Biological Chemistry*.

[21] Echtenkamp FJ, Zelin E, Oxelmark E (2011). Global functional map of the p23 molecular chaperone reveals an extensive cellular network. *Molecular Cell*.

[22] Karagöz GE, Duarte AMS, Ippel H (2011). N-terminal domain of human Hsp90 triggers binding to the cochaperone p23. *Proceedings of the National Academy of Sciences of the United States of America*.

[23] Cox MB, Miller CA (2004). Cooperation of heat shock protein 90 and p23 in aryl hydrocarbon receptor signaling. *Cell Stress and Chaperones*.

[24] Hollingshead BD, Petrulis JR, Perdew GH (2004). The aryl hydrocarbon (Ah) receptor transcriptional regulator hepatitis B virus X-associated protein 2 antagonizes p23 binding to Ah receptor-Hsp90 complexes and is dispensable for receptor function. *The Journal of Biological Chemistry*.

[25] Abbott BD, Probst MR, Perdew GH, Buckalew AR (1998). AH receptor, ARNT, glucocorticoid receptor, EGF receptor, EGF, TGF *α*, TGF *β* 1, TGF *β* 2, and TGF *β* 3 expression in human embryonic palate, and effects of 2,3,7,8-tetrachlorodibenzo-p-dioxin (TCDD). *Teratology*.

[26] Beischlag TV, Perdew GH (2005). ER*α*-AHR-ARNT protein-protein interactions mediate estradiol-dependent transrepression of dioxin-inducible gene transcription. *The Journal of Biological Chemistry*.

[27] Biegel L, Safe S (1990). Effects of 2,3,7,8-tetrachlorodibenzo-p-dioxin (TCDD) on cell growth and the secretion of the estrogen-induced 34-, 52- and 160-kDa proteins in human breast cancer cells. *Journal of Steroid Biochemistry and Molecular Biology*.

[28] Tappenden DM, Lynn SG, Crawford RB (2011). The aryl hydrocarbon receptor interacts with ATP5*α*1, a subunit of the ATP synthase complex, and modulates mitochondrial function. *Toxicology and Applied Pharmacology*.

[29] Schmidt JV, Bradfield CA (1996). AH receptor signaling pathways. *Annual Review of Cell and Developmental Biology*.

[30] Lowry OH, Rosebrough NJ, Farr AL, Randall RJ (1951). Protein measurement with the Folin phenol reagent. *The The Journal of Biological Chemistry*.

[31] Vengellur A, LaPres JJ (2004). The role of hypoxia inducible factor 1*α* in cobalt chloride induced cell death in mouse embryonic fibroblasts. *Toxicological Sciences*.

[32] Shevchenko A, Wilm M, Vorm O, Mann M (1996). Mass spectrometric sequencing of proteins from silver-stained polyacrylamide gels. *Analytical Chemistry*.

[33] Ohmura K, Kohno N, Kobayashi Y (1999). A homologue of pancreatic trypsin is localized in the acrosome of mammalian sperm and is released during acrosome reaction. *The Journal of Biological Chemistry*.

[34] Jia L, Kaur J, Stuart RA (2009). Mapping of the *Saccharomyces cerevisiae* oxa1-mitochondrial ribosome interface and identification of MrpL40, a ribosomal protein in close proximity to oxal and critical for oxidative phosphorylation complex assembly. *Eukaryotic Cell*.

[35] Accardi R, Oxelmark E, Jauniaux N, de Pinto V, Marchini A, Tommasino M (2004). High levels of the mitochondrial large ribosomal subunit protein 40 prevent loss of mitochondrial DNA in null mmfl *Saccharomyces cerevisiae* cells. *Yeast*.

[36] Maynard TM, Meechan DW, Dudevoir ML (2008). Mitochondrial localization and function of a subset of 22q11 deletion syndrome candidate genes. *Molecular and Cellular Neuroscience*.

[37] Funke B, Puech A, Saint-Jore B, Pandita R, Skoultchi A, Morrow B (1998). Isolation and characterization of a human gene containing a nuclear localization signal from the critical region for velo-cardio-facial syndrome on 22q11. *Genomics*.

[38] Auernhammer CJ, Isele NB, Kopp FB (2003). Novel neurotrophin-1/B cell-stimulating factor-3 (cardiotrophin-like cytokine) stimulates corticotroph function via a signal transducer and activator of transcription-dependent mechanism negatively regulated by suppressor of cytokine signaling-3. *Endocrinology*.

[39] Lelièvre E, Plun-Favreau H, Chevalier S (2001). Signaling pathways recruited by the cardiotrophin-like cytokine/cytokine-like factor-1 composite cytokine. Specific requirement of the membrane-bound form of ciliary neurotrophic factor receptor *α* component. *The Journal of Biological Chemistry*.

[40] Blot G, Lopez-Vergès S, Treand C (2006). Luman, a new partner of HIV-1 TMgp41, interferes with Tat-mediated transcription of the HIV-1 LTR. *Journal of Molecular Biology*.

[41] Lee MW, Chanda D, Yang J (2010). Regulation of hepatic gluconeogenesis by an ER-bound transcription factor, CREBH. *Cell Metabolism*.

[42] Adra CN, Donato J, Badovinac R (2000). SMARCAD1, a novel human helicase family-defining member associated with genetic instability: cloning, expression, and mapping to 4q22-q23, a band rich in breakpoints and deletion mutants involved in several human diseases. *Genomics*.

[43] Moldave K (1985). Eukaryotic protein synthesis. *Annual Review of Biochemistry*.

[44] Kahlert C, Weber H, Mogler C (2009). Increased expression of ALCAMCD166 in pancreatic cancer is an independent prognostic marker for poor survival and early tumour relapse. *The British Journal of Cancer*.

[45] Kulasingam V, Zheng Y, Soosaipillai A, Leon AE, Gion M, Diamandis EP (2009). Activated leukocyte cell adhesion molecule: a novel biomarker for breast cancer. *International Journal of Cancer*.

[46] Ofori-Acquah SF, King JA (2008). Activated leukocyte cell adhesion molecule: a new paradox in cancer. *Translational Research*.

[47] Song S, Kole S, Precht P, Pazin MJ, Bernier M (2010). Activation of heat shock factor 1 plays a role in pyrrolidine dithiocarbamate-mediated expression of the co-chaperone BAG3. *International Journal of Biochemistry and Cell Biology*.

[48] McCollum AK, Casagrande G, Kohn EC (2010). Caught in the middle: the role of Bag3 in disease. *Biochemical Journal*.

[49] Iwasaki M, Homma S, Hishiya A, Dolezal SJ, Reed JC, Takayama S (2007). BAG3 regulates motility and adhesion of epithelial cancer cells. *Cancer Research*.

[50] Rosati A, Di Salle E, Luberto L (2009). Identification of a Btk-BAG3 complex induced by oxidative stress. *Leukemia*.

[51] Hashimoto S, Hashimoto A, Yamada A, Onodera Y, Sabe H (2006). Assays and properties of the ArfGAPs, AMAP1 and AMAP2, in Arf6 function. *Methods in Enzymology*.

[52] Matsui C, Kaieda S, Ikegami T, Mimori-Kiyosue Y (2008). Identification of a link between the SAMP repeats of adenomatous polyposis coli tumor suppressor and the Src homology 3 domain of DDEF. *The Journal of Biological Chemistry*.

[53] Naz RK, Dhandapani L (2010). Identification of human sperm proteins that interact with human zona pellucida3 (ZP3) using yeast two-hybrid system. *Journal of Reproductive Immunology*.

[54] Kerkvliet NI (1995). Immunological effects of chlorinated dibenzo-p-dioxins. *Environmental Health Perspectives*.

[55] Vos JG, van Loveren H (1995). Markers for immunotoxic effects in rodents and man. *Toxicology Letters*.

[56] Puga A, Ma C, Marlowe JL (2009). The aryl hydrocarbon receptor cross-talks with multiple signal transduction pathways. *Biochemical Pharmacology*.

[57] Aly HA, Domènech O (2009). Cytotoxicity and mitochondrial dysfunction of 2,3,7,8-tetrachlorodibenzo-p-dioxin (TCDD) in isolated rat hepatocytes. *Toxicology Letters*.

[58] Alexander DL, Eltom SE, Jefcoate CR (1997). Ah receptor regulation of CYP1B1 expression in primary mouse embryo-derived cells. *Cancer Research*.

[59] Yao G, Craven M, Drinkwater N, Bradfield CA (2004). Interaction networks in yeast define and enumerate the signaling steps of the vertebrate aryl hydrocarbon receptor. *PLoS Biology*.

[60] Tian Y, Rabson AB, Gallo MA (2002). Ah receptor and NF-*κ*B interactions: mechanisms and physiological implications. *Chemico-Biological Interactions*.

[61] Roulhac PL, Ward JM, Thompson JW (2011). Microproteomics: quantitative proteomic profiling of small numbers of laser-captured cells. *Cold Spring Harbor Protocols*.

[62] Puri P, Acker-Palmer A, Stahler R, Chen Y, Kline D, Vijayaraghavan S (2011). Identification of testis 14-3-3 binding proteins by tandem affinity purification. *Spermatogenesis*.

[63] Agrawal P, Yu K, Salomon AR, Sedivy JM (2010). Proteomic profiling of Myc-associated proteins. *Cell Cycle*.

[64] Stohs SJ (1990). Oxidative stress induced by 2,3,7,8-tetrachlorodibenzo-p-dioxin (TCDD). *Free Radical Biology and Medicine*.

[65] Shertzer HG, Genter MB, Shen D, Nebert DW, Chen Y, Dalton TP (2006). TCDD decreases ATP levels and increases reactive oxygen production through changes in mitochondrial F0F1-ATP synthase and ubiquinone. *Toxicology and Applied Pharmacology*.

[66] Rosati A, Leone A, Del Valle L, Amini S, Khalili K, Turco MC (2007). Evidence for BAG3 modulation of HIV-1 gene transcription. *Journal of Cellular Physiology*.

[67] Lund AK, Goens MB, Kanagy NL, Walker MK (2003). Cardiac hypertrophy in aryl hydrocarbon receptor null mice is correlated with elevated angiotensin II, endothelin-1, and mean arterial blood pressure. *Toxicology and Applied Pharmacology*.

[68] Morris DL, Jeong HG, Jordan SD, Kaminski NE, Holsapple MP (1998). Characterization of the effects of 2,3,7,8-tetrachloredibenzo-p-dioxin in B6C3F1 and DBA/2 mice following single and repeated exposures. *Archives of Toxicology*.

[69] Sulentic CEW, Holsapple MP, Kaminski NE (1998). Aryl hydrocarbon receptor-dependent suppression by 2,3,7,8-tetrachlorodibenzo-p-dioxin of IgM secretion in activated B cells. *Molecular Pharmacology*.

